# *Aspergillus terreus* camptothecin-sodium alginate/titanium dioxide nanoparticles as a novel nanocomposite with enhanced compatibility and anticancer efficiency in vivo

**DOI:** 10.1186/s12896-023-00778-6

**Published:** 2023-04-01

**Authors:** Nermeen A. Kelany, Ashraf S. A. El-Sayed, Manar A. Ibrahim

**Affiliations:** 1 Department of Physics, Faculty of Science, Zagazig University, PO 44519, Zagazig, Egypt; 2 Enzymology and Fungal Biotechnology Lab, Botany and Microbiology Department, Faculty of Science, Zagazig University, Zagazig, 44519 Egypt

**Keywords:** Camptothecin, Sodium alginate, Titanium dioxide nanoparticles, Anticancer efficiency

## Abstract

**Background:**

Camptothecin derivatives are one of the most prescribed anticancer drugs for cancer patients, however, the availability, efficiency, and water solubility are the major challenges that halt the applicability of this drug.

**Methods:**

Biosynthetic potency of camptothecin by *Aspergillus terreus*, open a new avenue for commercial camptothecin production, due to their short-life span, feasibility of controlled growth conditions, and affordability for higher growth, that fulfill the availability of the scaffold of this drug.

**Results:**

Camptothecin (CPT) was purified from the filtrates of *A. terreus*, and their purity was checked by HPLC, and its chemical structure was verified by LC/MS, regarding to the authentic one. To improve the anticancer efficiency of *A. terreus* CPT, the drug was conjugated with sodium alginate (SA)/Titanium dioxide nanoparticles (TiO_2_NPs) composites, and their physicochemical properties were assessed. From the FT-IR profile, a numerous hydrogen bond interactions between TiO_2_ and SA chains in the SA/TiO_2_ nanocomposites, in addition to the spectral changes in the characteristic bands of both SA/TiO_2_ and CPT that confirmed their interactions. Transmission electron microscopy analysis reveals the spherical morphology of the developed SA/TiO_2_NPs nanocomposite, with the average particle size ~ 13.3 ± 0.35 nm. From the results of zeta potential, successful loading and binding of CPT with SA/TiO_2_ nanocomposites were observed.

**Conclusion:**

The in vivo study authenticates the significant improvement of the antitumor activity of CPT upon loading in SA/TiO_2_ nanocomposites, with affordable stability of the green synthesized TiO_2_NPs with *Aloe vera* leaves extract.

## Introduction

Camptothecin (CPT) and their derivatives are one of the most commercial broad-spectrum anticancer drugs for numerous types of malignancies, including melanoma, breast cancer, prostatic carcinoma, hepatocarcinoma, and colon carcinoma. The powerful anticancer activity of camptothecin elaborates from its specificity to inhibit with topoisomerase I (Topo I), thus, preventing DNA replication and RNA synthesis by stabilizing the Topo I-DNA complexes [1]. Commercially, camptothecin is the third largest commercial anticancer drug after Taxol and vincristine [1], however, the availability, poor water solubility [2], toxicity, and rapid plasma clearance are the major hurdles that limits their wide-spectrum applications. Biosynthetic potency of camptothecin by fungi being an affordable approach for commercial production of camptothecin and their derivatives [3–8], nevertheless, the poor water solubility, toxicity and rapid plasma clearance still the major limitation. Several strategies of drug delivery have been proposed to improve the efficiency and targetability of the drug.

Targeted drug delivery is one of the recent approaches for directing the drug to the diseased sites, improving the drug’s therapeutic index by facilitating their pass to the target sites, shielding the drugs from degrading enzymes, pH, facilitating the lower doses of drug to achieve highest therapeutic index [9]. Recently, nanoparticles have been recognized as a remarkable approach in the biomedical fields especially in drug delivery, cell labeling, tissue engineering, and controlled drug release [10]. Titanium dioxide nanoparticles (TiO_2_NPs) are transition metal oxides existing in a number of crystalline forms that has been recently used in various drug delivery applications (Venkatasubbu et al., 2013, 2015). Four common polymorphs of TiO_2_ exists in nature namely anatase (tetragonal), rutile (tetragonal), brookite (orthorhomic) and TiO_2_ (monoclinic) [11]. TiO_2_ is the ideal semiconductor for photocatalysis due to its high photo-activity, low cost, low toxicity, thermal stability, biocompatibility, and biological activity [6]. TiO_2_NPs have different physiochemical properties that affected not only by the crystal structure, and intrinsic electronic structure, but also by their shape, size, and doping [12, 13]. TiO_2_ attracted strong attention in drug delivery systems, especially with releasing the drug in a pH-dependent manner into the cells [14]. TiO_2_ have been used as a carrier for chemotherapeutic drugs such as Doxorubicin, Temozolomide, and Danuorubicin [15, 16], that displayed a higher efficiency than the corresponding free drugs. Mesoporous Titania nanoparticles with florescence properties have a high affinity to bind with the phosphate moieties of DNA, thus, it has been frequently used in DNA bioimaging [17]. In addition, the photocatalytic activity of TiO_2_ that helps in reducing the drug resistance, and provide cancer cell targeting [18].

TiO_2_ NPs have been synthesized by several approaches including physical methods; sputtering, low-pressure gas evaporation, plasma, high energy ball milling, and chemical methods by oxidation- reduction process, laser synthesis, hydrothermal and sol–gel methods, however, these methods are potentially hazardous, costly, requiring high energy [19]. Thus, the green synthesis approach using the plant extracts has been developed as an eco-friendly, economical safer, non-toxic and simpler route of synthesis of large scale TiO_2_NPs. Furthermore, the green synthesis approaches doesn't require high pressure, high temperature, hazards chemicals, in contrary to the physical and chemical methods [20]. Various plant extracts have been proved to be potential reducing agents, therefore, they could be successfully involved in the green synthesis of TiO_2_NPs [21].

Sodium alginate (SA) is an anionic polysaccharide composed of β-D-mannuronic acid and α-L-guluronic acid, easily cross-linked with divalent cationic linkers for various drug delivery applications. SA has been mostly used in biotechnology and biomedical applications due to their excellent properties, biocompatibility, biodegradability, non-toxicity, non-immunogenicity [22], anti-inflammatory [23], anticancer [24], and antioxidant activities [25]. Thus, the composite SA-TiO_2_ nanoparticles have a wide application in the tissue engineering fields [26], due to their enhanced cellular attachment [27]. Recently, SA-TiO_2_ has been used as a vehicle for simvastatin delivery with significant effect on primary human osteoblasts differentiation [28], and treatment of neuroblastoma [29]. Thus, the poor water solubility and rapid plasma clearance still the major limitations for further application of camptothecin. So, the objective of this study was to extract camptothecin from the cultures of *A. terreus,* and to conjugate the purified camptothecin with SA/TiO_2_ nanocomposite. As well as, the physicochemical properties and in vivo antiproliferative activity of the developed nanocomposite were characterized.

## Materials and methods

### Materials

Titanium tetrachloride (TiCl_4_), purity ≥ 99%, was purchased from Sigma-Aldrich Merck KGaA, Darmstadt Germany. The plant extract was prepared from healthy leaves of *Aloe vera* collected from the Botany department, Faculty of Science, Zagazig University. Sodium alginate (SA) was purchased from Loba Chemie, Mumbai, India. Double distilled (DD) water was purchased from Al-Gomhoria Company, Cairo, Egypt.

### Extraction and chemical validation of camptothecin from *Aspergillus terreus*

*Aspergillus terreus* was grown on dextrose broth (PDB) (200 g potato extract and 20 g glucose per liter) (BD, Difco, Cat# DF0549-17-9) [4–8], at 30 °C for 20 days. The cultures were filtered, and the filtrates were centrifuged at 5000 rpm, and the supernatant was used for camptothecin extraction by CHCl_3_: MeOH (4:1) [7, 8]. The organic phase containing camptothecin was concentrated by a rotary evaporator, and the extract was fractionated by TLC using Merck 1 mm (20 × 20 cm) pre-coated silica gel plates (TLC Silica gel 60 F254, Merck KGaA, Darmstadt, Germany), with the solvent system of chloroform: methanol (9:1, v/v) [5]. The plates were visualized by UV illumination at λ_254_ nm, and the putative CPT spots have the same color, and relative mobility of authentic one (Cat. 7689-0 3-4), were considered. Camptothecin was purified by preparative TLC, and their purity was assessed by HPLC (YOUNG In, Chromass, 9110 + Quaternary Pump, Korea) with using a RP-C18 column (Eclipse Plus C18 4.6 mm, 150 mm, Cat. #959963-902) with isocratic mobile phase methanol/water (60:40 v/v) at a flow rate 1.0 mL/min for 20 min, scanned by photodiode array detector (DAD). The chemical identity and concentration of the putative camptothecin were confirmed from retention time and peak area of authentic example at λ_360_ nm [8]. The chemical structure of the putative spots of CPT was resolved by the liquid chromatography-tandem mass spectrometry (LC–MS/MS) [6–8], performed on Thermo Scientific LCQ Deca mass spectrometer equipped with an electrospray source, in a positive-ion mode. The mobile phase A was water with 0.1% formic acid, and mobile phase B was acetonitrile with 0.1% formic acid. The sample were injected into Hypersil Gold aQ C18 column, and the elution system was a gradient of 2–98% mobile phase B over 30 min with flow rate of 0.2 ml/min. The electrospray ionization (ESI) source operated with a spray voltage of 4 kV and a capillary temperature of 250 ^◦^C. The ion trap was scanned from *m/z* 300 to 2000 in a positive-ion mode, and the mass scan sequence was recorded between 300 and 2000 Da. The chemical identity of the components was identified based on their mass spectra fragmentation pattern and retention time with NIST mass spectral library.

### Preparation of TiO_2_ NPs

**Fig. 1:**
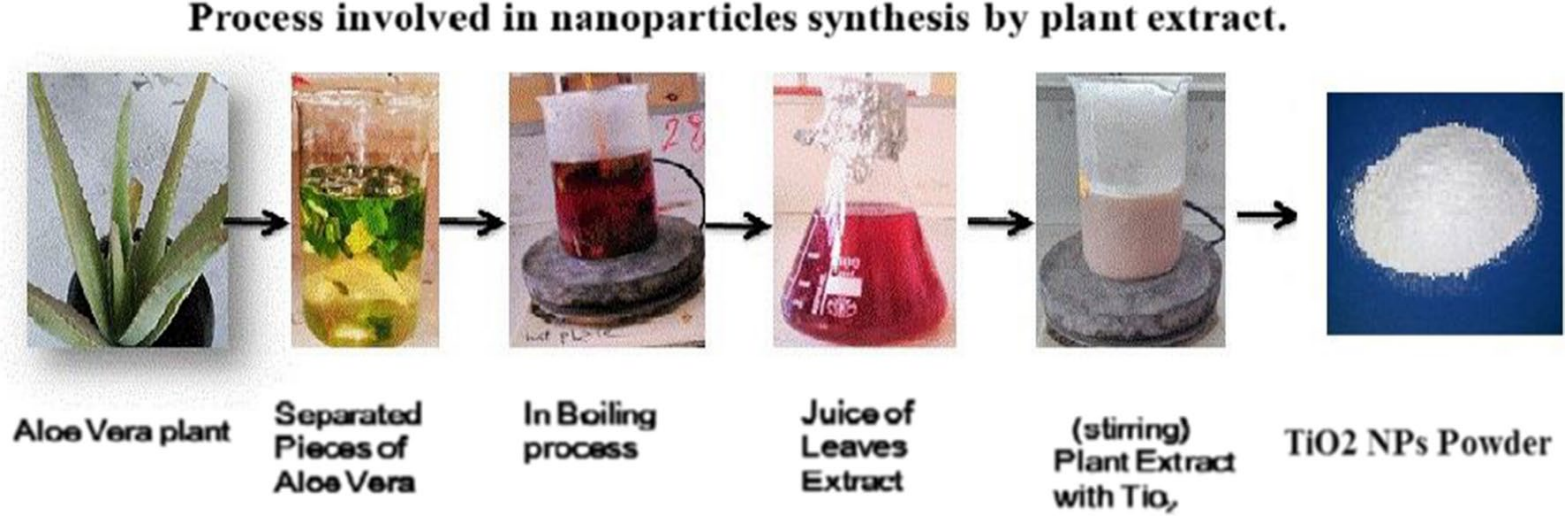
The scheme of green synthesis of TiO_2_NPs from the extract of *Aloe Vera*

Titanium dioxide nanoparticles (TiO_2_ NPs) were prepared at room temperature by eco-friendly green synthesis method using *Aloe Vera* leaves extract [30]. In brief, 100 ml of *Aloe vera* leaves extract was added drop wise to a 100 ml 1N TiCl_4_ solution in deionized water under continuous stirring. The pH of the mixture was adjusted at 9 and the stirring is continued at room temperature for 4 h. The developed nanoparticles were filtered, washed with double distilled water, and finally dried at 100 °C overnight. The obtained dry powder was further calcined at 500 °C for 4 h [30]. The overall scheme of green synthesis of TiO_2_NPs from extract of *A. vera* was shown in Fig. 1.

### Preparation of SA/TiO_2_ nanocomposite

Sodium alginate (SA) solution was prepared by dissolving 2 g of SA in 45 ml of double distilled water with constant stirring at 40 °C for 4 h until a clear solution was obtained. Separately, the pre- determined amount (0.22 g) of TiO_2_ nanoparticles was suspended in 5 ml double distilled water, homogenized in ultrasonic water bath (model CD-4820 170 W/42 kHz) for 30 min. The prepared TiO_2_NPs suspension was added to the viscous solution of SA with overnight continuous stirring at room temperature to produce uniformly dispersed SA/TiO_2_ solution.

### Preparation of CPT-SA/TiO_2_

**Fig. 2:**
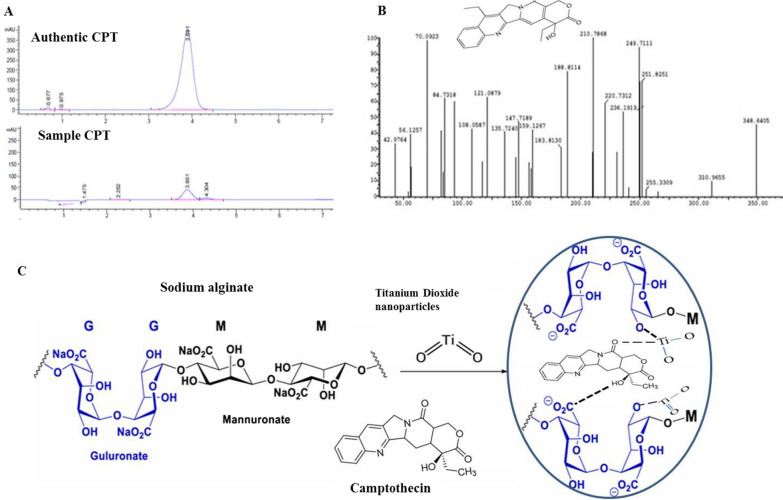
Chromatographic, spectroscopic analysis and chemical conjugation of camptothecin from the filtrates of *Aspergillus terreus.* After growing of the fungal culture, camptothecin was extracted and purified as described in Materials and Methods. The purity of extracted CPT of *A. terreus* was checked by HPLC (**A**), and its chemical structure was resolved from the LC/MS analysis (**B**). **C** Scheme of putative chemical conjugation of CPT-sodium alginate-titanium dioxide nanoparticles to form nanocomposite

The purified camptothecin from the cultures of *A. terreus* was checked by TLC and HPLC as described above, and then dissolved in 10% DMSO. The purified camptothecin preparations (100 µg/ml) were mixed with 50 mg SA/TiO_2_ in total volume 5 ml of double distilled water. The Scheme of preparations of CPT-SA/TiO_2_ was illustrated in Fig. 2. The composite of CPT-SA/TiO_2_ was incubated overnight at 4 °C with gentle stirring. The chemical and spectroscopic identities of the CPT-SA/TiO_2_ composites, as well as their biological activity in vivo and in vitro were assessed.

### Characterization techniques

#### Fourier transform infrared (FTIR)

FTIR spectra in the wavenumber range from 400 to 4000 cm^−1^ were recorded at room temperature for SA, TiO_2_NPs, CPT, SA/TiO_2_ and CPT-SA/TiO_2_ nanocomposites using FTIR spectrometer model (JASCO, FT/IR-4100 type A).

#### Transmission electron microscopy (TEM)

The structural morphology of the samples was investigated by high resolution transmission electron microscopy (HRTEM, JEOL JEM-1230). For HRTEM analysis, the prepared NPs and its composites were dispersed in ethanol with the help of ultra-sonication for 15 min and then loaded on a carbon-coated copper mesh.

#### Zeta potential

The zeta potential of TiO_2_ NPs, SA/TiO_2_ and CPT-SA/TiO_2_ nanocomposites were measured by dynamic light scattering techniques (Zeta sizer, Malvern, UK). The samples were diluted 1:100 by double distilled water and the measurements were done at 25 °C and detection angle 90°.

### In vivo study

#### Animals and tumor implantation

Thirty females, 7-week old, albino mice of average weight 23 ± 3 g were housed in six clear plastic cages at room temperature (27 ± 3 °C) with a regular light/dark cycle and free access to food and water. The animals were provided from, and housed in, the animal house of the National cancer institute, Cairo University, Egypt. After being anesthetized with ketamine/ xylazine (100–200 mg/kg) [31], each animal was injected in the thigh of the hind limb with 2 × 10^6^ Ehrlich ascites carcinoma cells in 0.2 ml normal saline (collected from the peritoneal cavity of Ehrlich's ascites carcinoma mice, kindly supplied by the National Institute of Cancer Research, Cairo University).

The treatment protocol began 10 days post tumor implantation when the average tumor volume reached approximately 60 mm^3^. The animals were divided randomly into six groups, five animals each, as following: Group 1: animals injected with 0.05 ml of CPT solution, Group 2: animals injected with SA/TiO_2_ nanocomposite with high dose (0.022 ml), Group 3: animals injected with low dose (0.011 ml) of SA/TiO_2_ nanocomposite. Group 4: animals were injected with high dose (0.2 ml) of CPT-SA/TiO_2_ nanocomposite and Group 5: animals were injected with low dose (0.1 ml) of CPT-SA/TiO_2_ nanocomposite and Group 6 (control group): without treatment. All injections were done intra-tumoral at a dose equivalent to 2.5 mg CPT/kg body weight [32].

#### Assessment of treatment

A Vernier Caliper was used to measure the tumor's length and width for four weeks. The measurements were done at the same exact time of the day each time during the experiment. The tumor volume was computed using the following equation [33]:

$${\text{Tumor}}\;{\text{volume}} = 0.{5} \times \left( {{\text{d}}_{{1}} } \right)^{{2}} \times {\text{d}}_{{2}}$$

where d_1_ and d_2_ are the tumor width and length, respectively. The percentage of surviving animals in each group was determined daily and plotted as a function of days post-treatment. On day 30 post-treatment, the experiment was terminated; and an animal from each group was sacrificed by cervical dislocation. The tumor tissues were excised, fixed in 10% formalin, and embedded in paraffin. Sections were cut at a thickness of 3 μm, stained with hematoxylin and eosin (H&E), and then pathologically inspected under Olympus BX51 Microscope with Digital Camera to investigate the histopathological changes.

### Statistical analysis

The data were analyzed using SPSS software (SPSS, Inc., Chicago, Illinois, USA). All the results are presented as the mean of at least three attempts ± standard deviation. The two-way ANOVA test was used for data analysis followed by post hoc Tukey HSD test of significance, and a *p*-value of less than 0.05 was considered to be statistically significant (*p* < 0.05).

## Results and discussion

### Extraction, chemical validation and preparation of camptothecin-sodium alginate/TiO_2_ nanocomposite (CPT-SA/TiO_2_ conjugates)

Camptothecin has been extracted from the filtrates of *Aspergillus terreus*, purified by the preparative TLC, and the purity and its concentration were assessed by HPLC [4–6]. From the TLC and HPLC profile, the extracted putative camptothecin gave the same mobility time and color of authentic camptothecin. From the HPLC chromatogram (Fig. 2A), the purified camptothecin of *A. terreus* displayed a single sharp peak with retention time 3.91 min, comparing to the authentic one (3.87 min) ensuring its chemical proximity as camptothecin. The chemical structure of the extracted *A. terreus* camptothecin has been confirmed by LC–MS/MS (Fig. 2B). The putative camptothecin of *A. terreus* had the same molecular mass to charge ratio (348.2 m/z), in addition to the same molecular fragmentation pattern of authentic camptothecin of *Camptotheca acuminata* [6]. From the profile of the first mass spectra, a peak at retention time 10.38 min with a molecular ion peak at m/z 349 [M + H] + corresponding to the molecular formula C_20_H_16_N_2_O_4_ in addition to other diagnostic peaks of camptothecin alkaloid. The purified camptothecin from *A. terreus* was conjugated with sodium alginate/TiO_2_-nanocomposite according to the proposed scheme (Fig. 2C). The physiochemical properties of the CPT-SA/TiO_2_ nanocomposites were verified from the spectroscopic studies.

### FTIR spectroscopy analysis

**Fig. 3:**
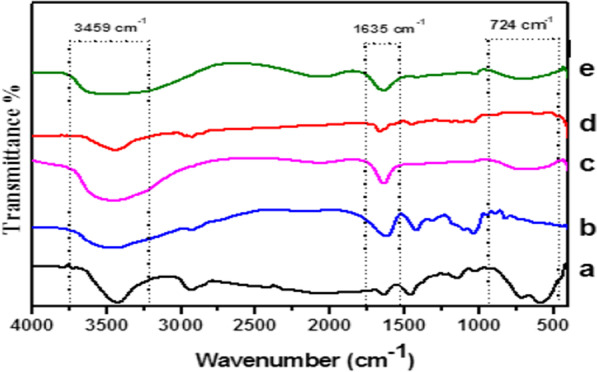
FT-IR spectra of TiO_2_NPs (**a**), SA (**b**), SA/TiO_2_ nanocomposite (**c**), CPT (**d**) and CPT-SA/TiO_2_ nanocomposite (**e**)

Chemical composition and possible interactions between TiO_2_ NPs, SA biopolymer matrices and camptothecin (CPT) were evaluated by FTIR spectroscopy. The characteristic spectrum of the sodium alginate biopolymer is composed of a broad band centered at approximately 3460 cm^−1^ that arises from the stretching of hydroxyl groups, low intensity bands at about 2922 cm^−1^ attributed to –CH_2_ groups as shown in Fig. 3. The assignments of the FT-IR absorption bands of SA, TiO_2_NPs, SA/TiO_2_, CPT and CPT-SA/TiO_2_ conjugates were shown in Table 1. The emerged band at 2164 cm^−1^ referred to CO_2_ group, while, the characteristic bands at 1620 and 1418 cm^−1^ referred to carboxylate salt group (-COONa) with asymmetric and symmetric stretching vibrations, respectively [34]. The observed peak at 1305 cm^−1^ refers to the asymmetrical stretching C–O group. Stretching vibrations of C–O–C was appeared at 1030 cm^−1^. Bands at 900–600 cm^−1^ were designated to the C–H bond stretching vibrations [35].

**Table 1:** Assignments of the FT-IR absorption bands of SA, TiO_2_NPs, SA/TiO_2_, CPT and CPT-SA/TiO_2_

Sample	Wavenumber (cm^−1^)	Assignments
SA	3460	OH stretching
	2922	CH_2_ asymmetric stretching
	1620	(–COONa) asymmetric stretching
	1418	(–COONa) symmetric stretching
	1305	C–O asymmetrical stretching
	1030	C–O–C stretching vibrations
	900–600	C–H stretching vibrations
TiO_2_ NPs	3435	OH stretching
	2928	C–H stretching vibrations
	1631	OH bending vibration of adsorbed water molecules
	1383	Ti–O–Ti stretching vibrations
	555	Ti–O stretching vibrations
SA/TiO_2_	3440	OH stretching
	1635	(–COONa) asymmetric stretching
	1418	(–COONa) symmetric stretching
	1030	C–O–C stretching vibrations
	670	Ti–O stretching vibrations
CPT	3438	OH stretching
	1666	C=O stretching
	1444	C=N stretching
	1113	C–C(=O)–O stretching
	1035	C–O stretching
	613	Hydrogen bonds on the heteroaromatic nucleus
CPT-SA/TiO_2_	3459	OH stretching
	1635	(–COONa) asymmetric stretching
	724	Ti–O stretching vibrations

From the FT-IR spectra of the prepared TiO_2_NPs (Fig. 3), the spectrum showed band centered at 3435 cm^−1^ corresponding to the stretching vibration of hydroxyl groups, while the band at 1631 cm^−1^ reveals the bending vibration of O–H bonds of adsorbed water molecules on the nanoparticles surface [36]. The band at 2928 cm^−1^ reveals stretching vibrations of C–H bonds, while, the peak centered at 555 cm^−1^ and 1383 cm^−1^ are due to the vibration of Ti–O and Ti–O–Ti bonds, respectively, in the TiO_2_ lattice.

After encapsulation of TiO_2_ NPs in SA matrix, there is an enlargement of the hydroxyl groups band and its center was displaced to 3440 cm^−1^. This indicates an increase of the hydrogen bond interactions between TiO_2_ and SA chains in the SA/TiO_2_ nanocomposite. The band observed at 2164 cm^−1^ due to CO_2_ group in SA shifted to 2062 cm^−1^. Furthermore, the intensity of band at 1620 cm^−1^ decreased and shifted to 1635 cm^−1^ in SA/TiO_2_ nanocomposite. Besides, there is increase in the intensity of the symmetric stretching mode of carboxylate salt groups (–COONa) located at 1418 cm^−1^. The intensity of the band characteristic to stretching vibrations of C–O–C at 1030 cm^−1^ in SA decreased in SA/TiO_2_ nanocomposite. There is a broad band located at 670 cm^−1^ which may be due to superimpose of C–H and Ti–O bonds stretching vibrations of SA and TiO_2_ NPs, respectively. These spectral changes clearly point out the presence of interfacial interactions between TiO_2_ NPs and SA components.

The FTIR spectra of camptothecin (CPT) (Fig. 3), shows the principal absorption bands of –OH stretching at 3438.46 cm^−1^, C=O stretching at 1666.2 cm^−1^, C=N at 1444.42 cm^−1^, C–C(=O)–O stretching at 1113.69 cm^−1^, C–O at 1035.59 cm^−1^ and band at 613.252 cm^−1^ that appears to be a contribution of four adjacent hydrogen bonds on the hetero-aromatic nucleus. For CPT-SA/TiO_2_ spectrum, characteristic structural bands of both SA/TiO_2_ and CPT were observed. The –OH stretching appeared at 3459.6 cm^−1^ and became broader as shown in Fig. 3 that could be due to an interaction with CPT. Also, the band at 1635 cm^−1^ became broader, Ti–O characteristic band at 675 cm^−1^ was shifted to 724 cm^−1^ due to physical interaction between TiO_2_ and CPT. These observations confirming the interaction between SA/TiO_2_ and CPT.

### Transmission electron microscopy analysis

**Fig. 4:**
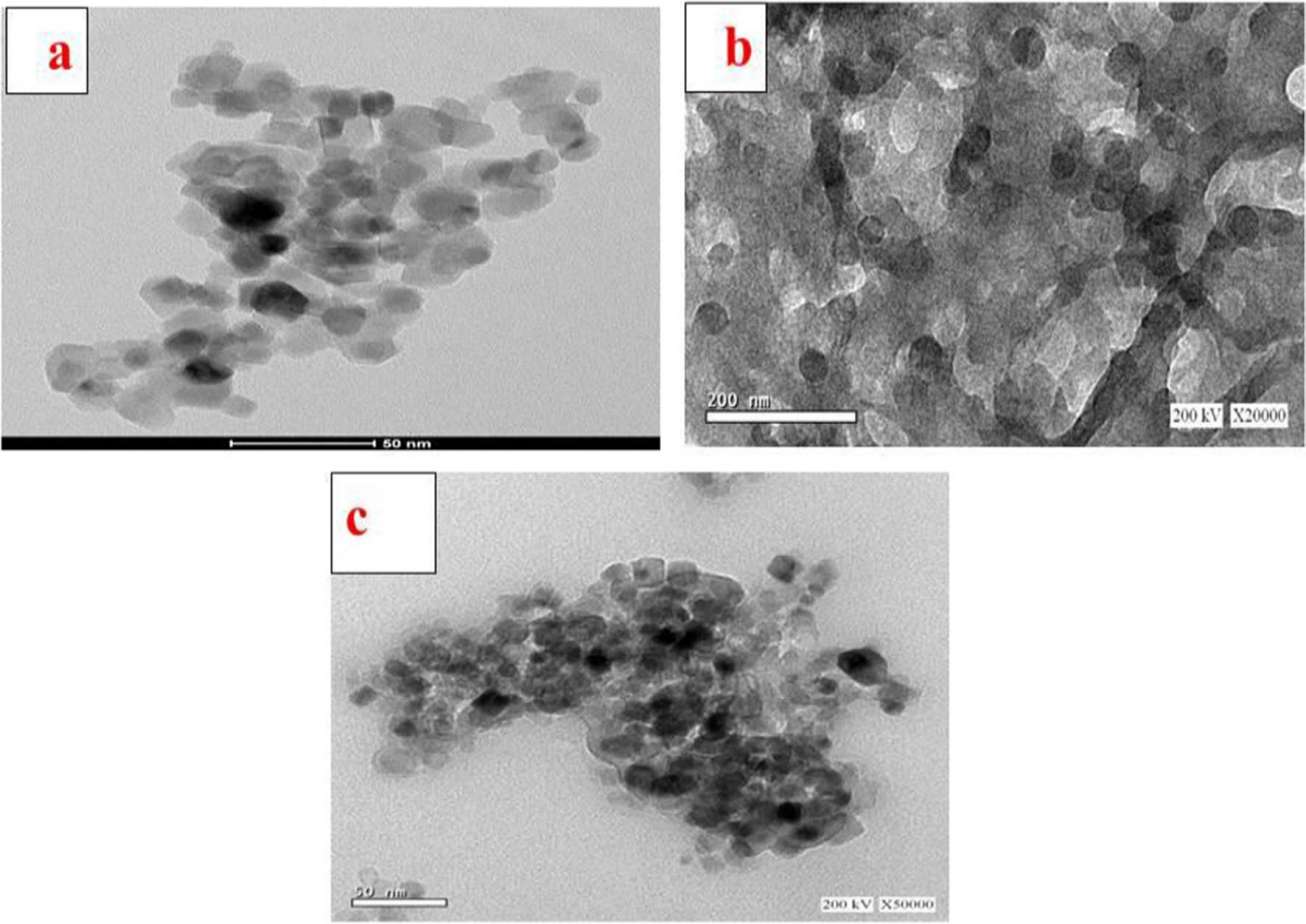
Transmission electron micrograph of TiO_2_NPs (**A**), SA/TiO_2_ nanocomposite (**B**) and CPT-SA/TiO_2_ nanocomposite

The morphology and size of the prepared CPT-SA-TiO_2_ nanocomposite were determined from the TEM analysis. The TiO_2_NPs possess a spherical morphology (Fig. 4a), with particle size of 13.3 ± 0.35 nm, nanoparticles with smaller sizes (< 200 nm) can greatly reduce the level of reticulo-endothelial system (RES) uptake and provide passive tumor targeting ability via the enhanced permeability and retention effect [37, 38]. TEM image of SA/TiO_2_ nanocomposite clearly showed the TiO_2_NPs with particle size of 36.23 ± 2.3 nm embedded and dispersed finely inside the matrix of SA due to the appearance of dark spheres inside the matrix and some incorporated nanospheres were attracted to the surface of matrix with no sign of agglomeration. On the other hand, sodium alginate with brighter color can be observed (Fig. 4b) which acting as a binder for the TiO_2_NPs. Due to the binding effect of alginate, clusters of TiO_2_ nanoparticles/alginate have formed which increases the effective size of the particles, supporting the success of SA/TiO_2_ nanocomposite preparation. TEM image of CPT-SA/TiO_2_ nanocomposite is shown in Fig. 4c. The image revealed dark and sphere-shaped particles without aggregation. The particles had homogeneous particle sizes with an average size of approximately 15.09. ± 2.84 nm.

### Zeta potential measurements

**Fig. 5:**
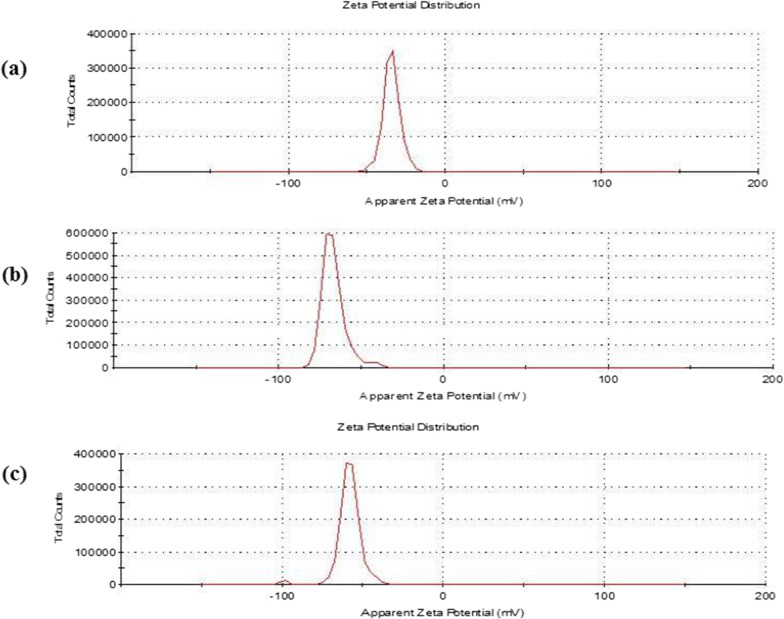
Zeta potential spectrum of TiO_2_NPs (**A**), SA/TiO_2_ nanocomposite, and CPT-SA/TiO_2_ nanocomposite (**C**)

The zeta potential (ZP) of the TiO_2_ nanoparticles, SA/TiO_2_ and CPT-SA/TiO_2_ nanocomposite was evaluated to estimate the colloidal stability of the NPs. The zeta potential of TiO_2_ nanoparticles were shown in Fig. 5a. The prepared TiO_2_ nanoparticles had large zeta value (− 34.1 ± 5 mV) indicating their greater stability in aqueous solutions, suggesting the repelling of the nanoparticles and low possibility to agglomerate [39, 40]. The zeta potential in the limit of ± 30 mV is an important parameter that reflects the nanoparticles stability [41]. TiO_2_ nanoparticles dispersed in water have negatively charged surface at neutral pH. It has been reported that presence of negative charge on the surface of particles can promote cell adhesion and proliferation [42].

The measured ZP for the suspension containing SA/TiO_2_ nanocomposite in a distilled water was highly negative − 66.8 ± 6.8 mV (Fig. 5b), as predictable, because alginate is well known for its anionic nature. It was noted that the ZP value of the two-component suspension was found to be lower than that for the suspension containing only Tio_2_, whereas CPT-SA/TiO_2_ had zeta potential of − 58.4 ± 6 mV (Fig. 5c). After CPT loading and binding, the significantly changed colloidal stability of the nanoparticles indicates the success of drug loading on the carrier. This increase in the zeta potential was attributed to attractive forces between CPT and SA/TiO_2_ by intermolecular interactions. The obtained results from zeta potentials were also direct evidence to confirm the successful loading and binding of CPT with SA/TiO_2_. The uptake and internalization of the negative charged nanoparticles was attributed to formation of double electrical layers on the nanoparticles surface with the dispersion on fluid. These surface two layers of charges surrounds the nanoparticles, which the internal “Stern” layer of positive charge form a bulk electrolytes bound to the nanoparticles, and the outer diffused layer with free ions bound to the particle electric surface potential. So, the surface charge of the nanoparticle interacting with dissolved ions in the bulk dispersant induces an electrical neutralization, thus, Zeta potential is rigorously not equal to the electric surface potential [43]. Consistently, a preferential uptake of the negatively charged cerium oxide nanoparticles by lung adenocarcinoma cells has been observed, due to the presence of frequent of patchy areas with cationic sites allowing the binding of the negatively charged nanoparticles in clusters followed by subsequent endocytosis [44, 45].

### In vivo study

#### Tumor size measurements

**Fig. 6:**
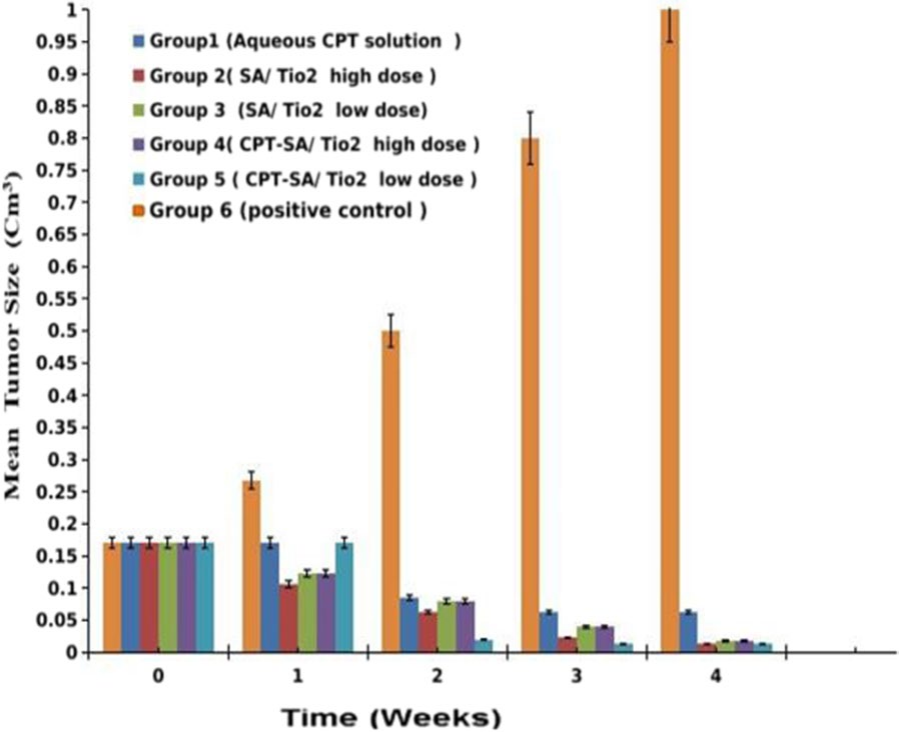
The mean tumor volume as a function of the time post-treatment

The mean tumor volume of different groups along four weeks post treatment showed that the treated groups experienced significant inhibition of tumor growth compared to positive control group (*p* < 0.05). The tumor volume in group 4 (CPT-SA/TiO_2_ nanocomposites with high dose), group 2 (SA/TiO_2_ nanocomposites with high dose) and group 3 (CPT-SA/TiO_2_ nanocomposites with low dose) was dramatically decreased along the four weeks with no significant difference between them as shown in Fig. 6. However, the mean tumor volume of them was significantly lower than that of groups 5 and 1 (received SA/TiO_2_ nanocomposites with low dose and aqueous CPT solution, respectively) (*p* < 0.05).

#### Survival assay

**Fig. 7:**
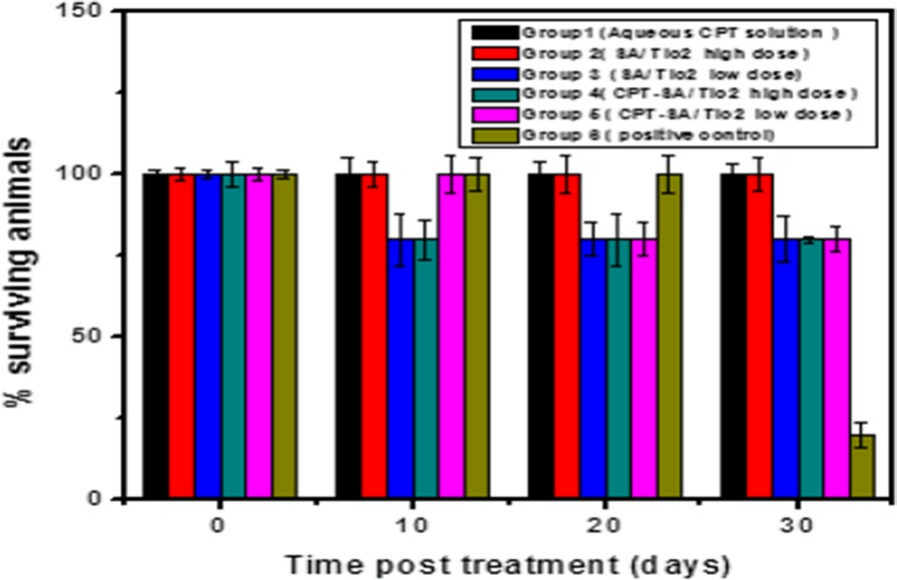
The percentage of surviving animals in the different groups as function of incubation time following tumor implantation

To assess the anti-tumor activity in vivo, CPT loaded in SA/TiO_2_ nanocomposites (CPT-SA/TiO_2_ nanocomposites) was directly injected to the intratumoral in albino mice [46]. The antitumor activity of aqueous CPT solution was compared to SA/TiO_2_ and CPT-SA/TiO_2_ nanocomposites with. These four groups were compared to positive control group (without treatment). The day of death of each mouse from each group was recorded and the percent of surviving animals was calculated. The experiment was terminated 30 days post treatment. Figure 7 shows the variation of survival animal percentage as a function of incubation time following tumor implantation. At 30 days post treatment, 20, 80, 80, and 80% of animals in positive control, SA/TiO_2_ nanocomposites with low dose, CPT-SA/TiO_2_ nanocomposites with low dose and CPT-SA/TiO_2_ nanocomposites with high dose groups, respectively were survived. However, 100% of mice injected by aqueous CPT solution and SA/TiO_2_ nanocomposites with high dose were still alive. The results of survival assay revealed that the mice, which received aqueous CPT solution and SA/TiO_2_ nanocomposites with high dose groups have survived for longer time compared to the other groups. These results were confirmed by the histopathological examination.

#### Histopathological examination

Histopathological examination was done to investigate pathological changes that occurred to the tumor as a response to the chemotherapeutic treatment [47]. Histopathological examinations confirmed the observed inhibition of tumor growth rate for treated groups in addition to the high growth rate of the positive control group. Examination of the entire tumor sections for the various groups revealed marked differences in cellular features accompanied by varying degrees in necrosis percentage.

**Fig. 8:**
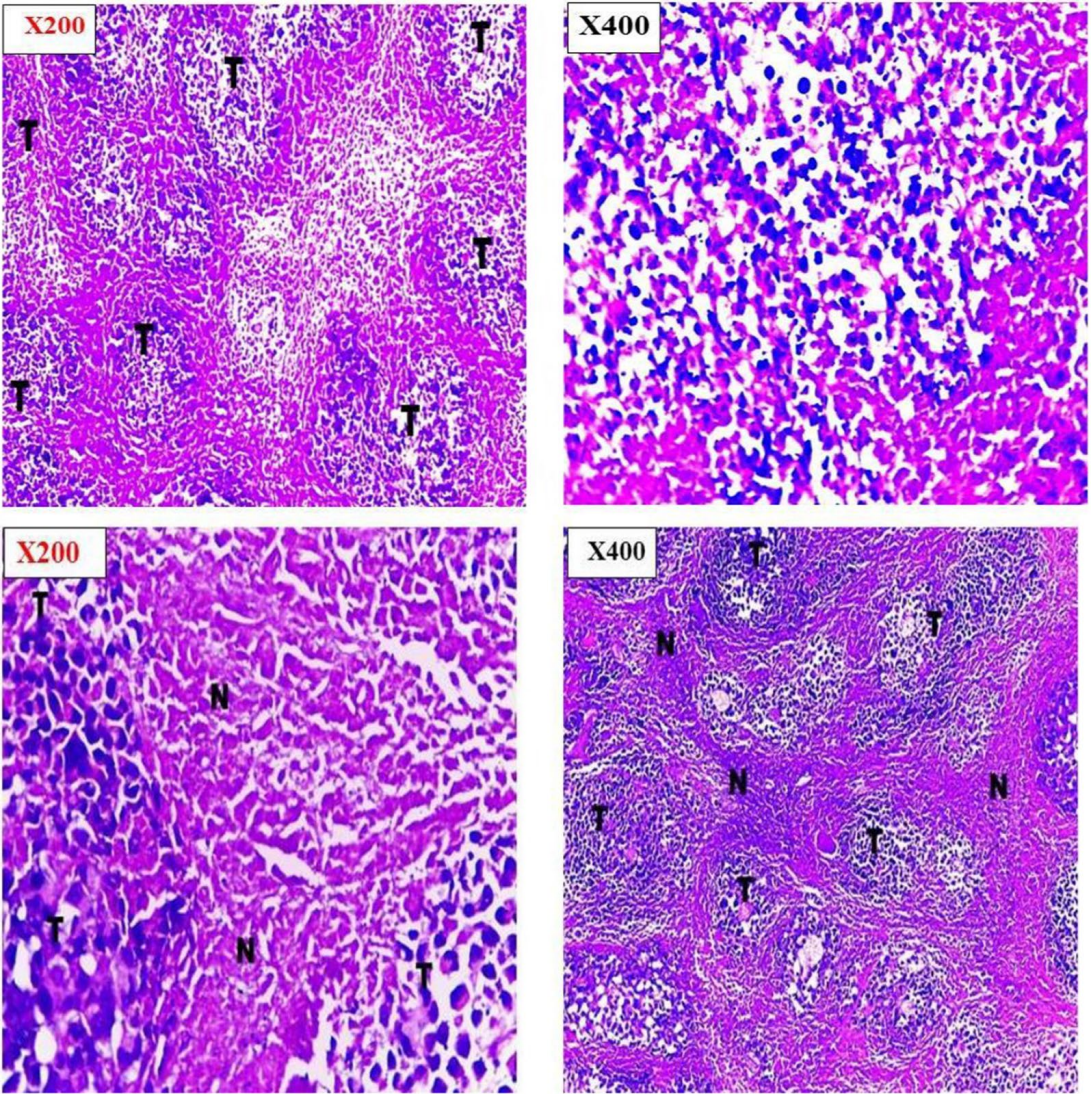
Histopathological sections of negative control of Ehrlich ascites carcinoma and CPT treated Ehrlich ascites carcinoma at 200 × and 400 × by Olympus BX51 Microscope

**Fig. 9:**
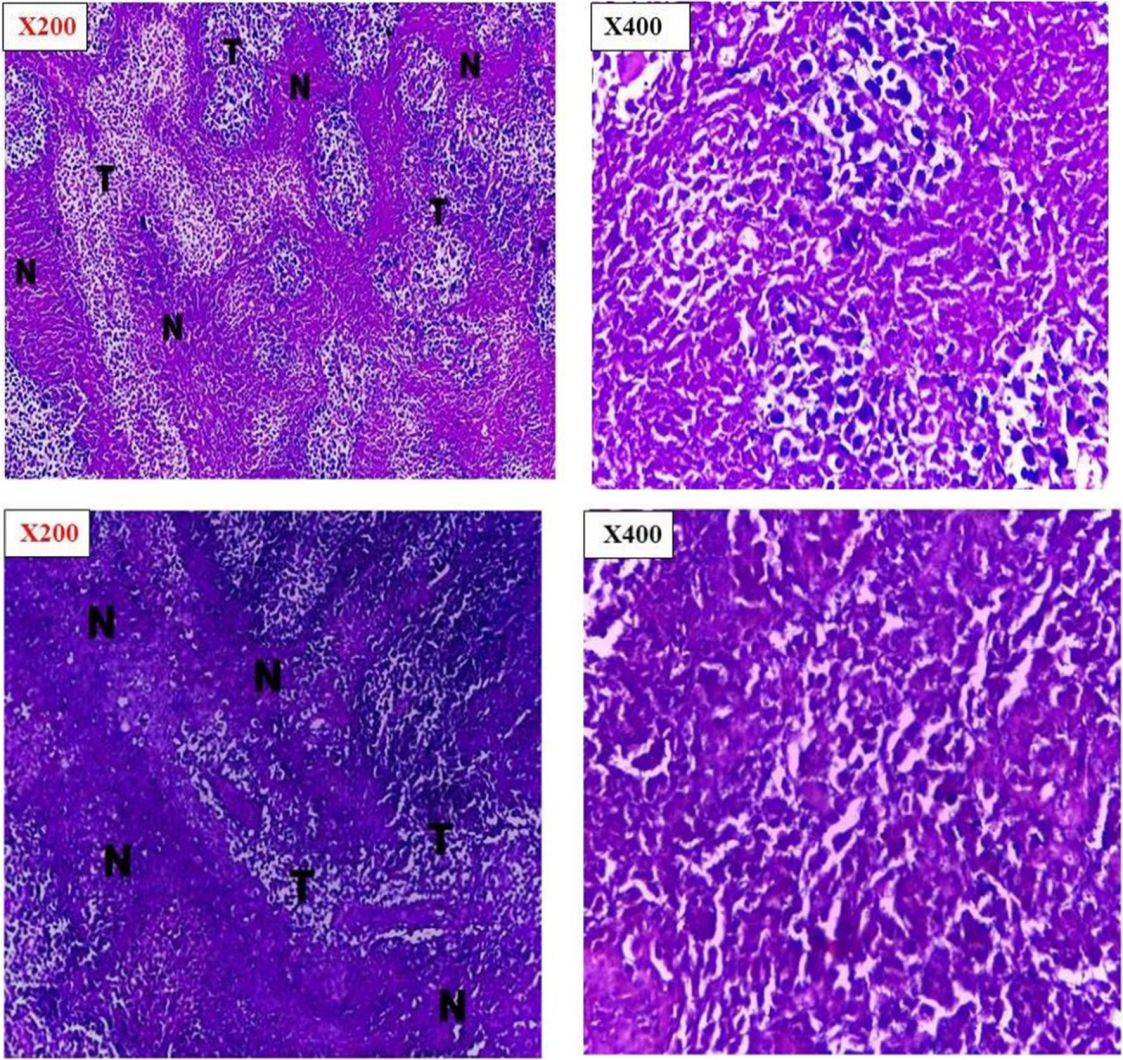
Histopathological sections of SA/TiO_2_ nanocomposite treated Ehrlich ascites carcinoma at 200 × and 400 × , showing different degree of necrosis

**Fig. 10:**
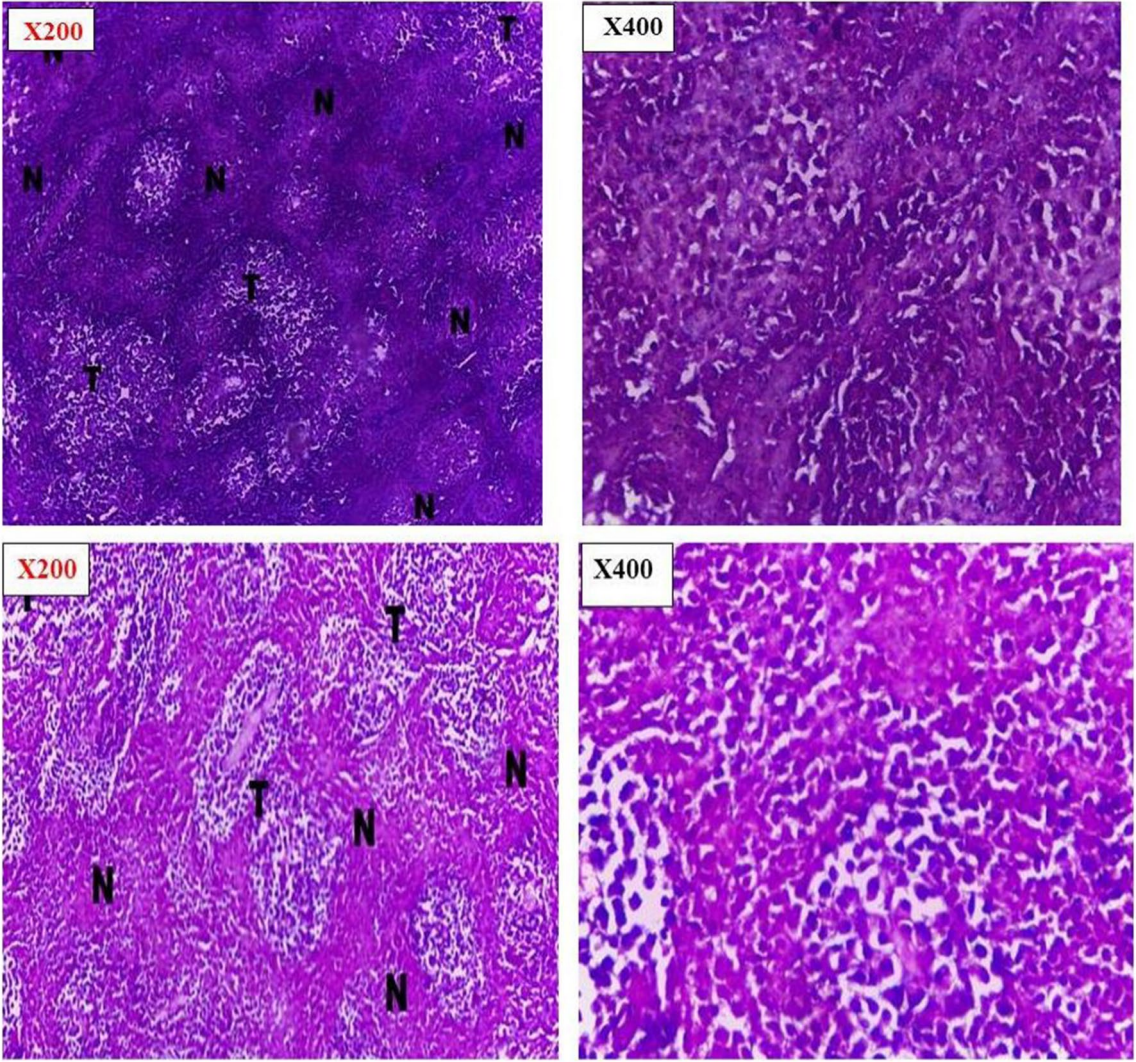
Histopathological sections of CPT-SA/TiO_2_ treated Ehrlich ascites carcinoma at 200 × and 400 × , showing different degree of necrosis at lower dose and higher dose

Tumor sections taken from the last animal died in control group showed well defined tumor tissue that characterized by proliferation of malignant epithelial cells which is pleomorphic, hyper- chromatic nuclei with mitotic as shown in Fig. 8. While tumor sections from aqueous CPT solution and SA/TiO_2_ nanocomposites with low dose groups showed little necrosis within wide areas of viable tumor cells (tumor necrosis index about 63.4% and 77.8% respectively) were observed in Figs. 8 and 9. The areas of necrosis and fibrosis with few scattered degenerated tumor cells for CPT-SA/TiO_2_ nanocomposites with low dose, SA/TiO_2_ nanocomposites with high dose, and CPT-SA/TiO_2_ nanocomposites with high dose groups (tumor necrosis index about 92.1%, 87.3%, and 85.4%, respectively) (Fig. 10). The combined observations from tumor volume and histopathology confirmed that the antitumor activity of CPT was significantly improved when loaded in SA/TiO_2_ nanocomposites where TiO_2_ NPs was successfully synthesized by green synthesis method using *Aloe Vera* leaves extract.

In conclusion, camptothecin was purified from *A. terreus*, their chemical structure was verified, conjugated with SA/TiO_2_NPs composites, and the physicochemical properties and the in vivo antiproliferative activity of the CPT/SA/TiO_2_NP nanocomposite were assessed. The efficiency of the extracted *A. terreus* CPT towards the Ehrlich ascites carcinoma was strongly improved upon conjugation with SA/TiO_2_NPs nanocomposite, as revealed from the in vivo pathological analyses.

## Data Availability

The datasets of the current study are available from the corresponding author on a reasonable request.
